# Impact of Exercise in Community-Dwelling Older Adults

**DOI:** 10.1371/journal.pone.0006174

**Published:** 2009-07-08

**Authors:** Ruth E. Hubbard, Nader Fallah, Samuel D. Searle, Arnold Mitnitski, Kenneth Rockwood

**Affiliations:** 1 Geriatric Medicine Research Unit, Dalhousie University and Queen Elizabeth II Health Sciences Centre, Halifax, Canada; 2 Division of Geriatric Medicine, Department of Medicine, Dalhousie University, Halifax, Canada; University of Toronto, Canada

## Abstract

**Background:**

Concern has been expressed that preventive measures in older people might increase frailty by increasing survival without improving health. We investigated the impact of exercise on the probabilities of health improvement, deterioration and death in community-dwelling older people.

**Methods and Principal Findings:**

In the Canadian Study of Health and Aging, health status was measured by a frailty index based on the number of health deficits. Exercise was classified as either high or low/no exercise, using a validated, self-administered questionnaire. Health status and survival were re-assessed at 5 years. Of 6297 eligible participants, 5555 had complete data. Across all grades of frailty, death rates for both men and women aged over 75 who exercised were similar to their peers aged 65 to 75 who did not exercise. In addition, while all those who exercised had a greater chance of improving their health status, the greatest benefits were in those who were more frail (e.g. improvement or stability was observed in 34% of high exercisers versus 26% of low/no exercisers for those with 2 deficits compared with 40% of high exercisers versus 22% of low/no exercisers for those with 9 deficits at baseline).

**Conclusions:**

In community-dwelling older people, exercise attenuated the impact of age on mortality across all grades of frailty. Exercise conferred its greatest benefits to improvements in health status in those who were more frail at baseline. The net effect of exercise should therefore be to improve health status at the population level.

## Introduction

The benefits of exercise have long been recognized. Joseph Addison wrote in 1711 that without exercise “the body cannot subsist in its vigor, nor the soul act with cheerfulness” [1]. Exercise programmes of varying design have diverse positive effects in community-dwelling older people including improved muscle strength and gait speed [2], reduction in falls [3] improved balance [4] and increased bone mineral density [5]. In longitudinal cohort studies, physical activity is protective of impaired physical function [6] and modifies the effect of disability on depression [7].

Exercise programmes for frail older people, however, have yielded conflicting results. A systematic review of physical training in institutionalised elderly indicated positive effects on muscle strength but effects on gait, disability, balance and endurance were inconclusive [8]. In some studies, exercise programmes in very frail older people result in no improvements in physical health or function [9] and increase musculoskeletal injury [10] and falls [11]. In contrast, other studies conclude that exercise improves physical performance scores [12] and reduces falls [13]. In an international observational study, physical activity in frail older people seemed to slow further functional decline [14].

The concern that preventive care in older people merely creates a different set of health problems has been expressed with some vigour both in the lay [15] and in the medical press [16] (e.g. “…preventive interventions are encouraged regardless of age, and thus can be harmful to the patient and expensive to the health service” [16]. Since exercise is associated with increased longevity [17] and frailty is inextricably linked with aging [18] exercise could, in theory, increase the overall burden of frailty by allowing more people to live to advanced old age where frailty is most common.

How exercise affects the health of older people over the long term is unlikely to be the subject of a randomized, controlled trial, given the many benefits known to be associated with physical activity in community-dwelling older people. In consequence, longitudinal, observational studies are essential if we are to understand whether the benefits of exercise extend to all older people, regardless of their frailty status or whether there is a certain age or physiological threshold beyond which exercise may not have a positive effect [19].

The aims of this study were to examine how exercise impacts the health of older people and how these impacts might differ by individual health status – i.e. by level of fitness or frailty. We also aimed to discriminate whether effects are due to slower decline, more frequent improvement, or differing mortality rates.

## Methods

### Ethics Statement

The Canadian Study of Health and Aging was approved by each of the Research Ethics Committees at the 36 participating centers. Approval for these analyses came from the Research Ethics Committee of the Capital District Health Authority, Halifax, Canada.

This is a secondary analysis of the Canadian Study of Health and Aging (CSHA), a nationally representative cohort study of people age 65 years and over at baseline [20]. Briefly, 9008 community-dwelling elderly people were randomly sampled from 36 communities in all 10 Canadian provinces. In this study, we examined the 6297 participants able to fully complete a self-administered risk factor questionnaire, investigating their frailty status at baseline (CSHA-1, conducted in 1990–1991) and at 5-year follow up (CSHA-2). Decedent information was collected at follow-up to assess date of death.

**Figure 1:**
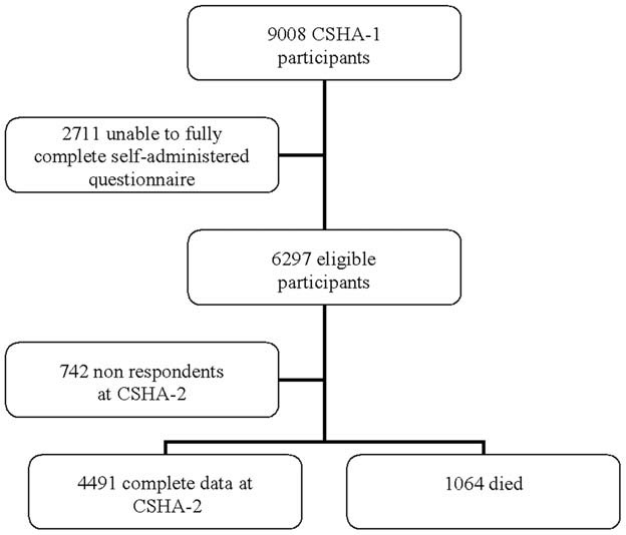
Derivation of Cohort.

The risk factor questionnaire addressed demographics, health attitudes, medical and family histories, activities of daily living and current health problems. Two questions based on the frequency and intensity of exercise assessed the level of physical activity, as validated elsewhere [21]. Subjects were classified as participating in ‘high exercise’ (three or more times per week, at least as intense as walking) or low/no exercise (all other exercisers and non exercisers). Of all 6297 eligible participants, 742 were lost to follow up. People with known frailty status at CSHA-2 (n = 4491) or those who died between CSHA-1 and CSHA-2 (n = 1064) were included in our sample (Figure 1).

### Frailty Index

In general, frailty is understood as an increased vulnerability to a range of adverse outcomes, including death, institutionalisation and worse health [22]. It can be operationalized in many ways. A variety of tools identify phenotypic frailty as a clinical syndrome (a set of signs and symptoms that tend to occur together, thus characterizing a specific medical condition) [23]. The most well known of these is Fried et al. 's frailty phenotype identifying someone as frail when they meet ≥3 of 5 criteria (unintentional weight loss of 10 lbs or more in past year, self reported exhaustion, weak grip strength, slow walking speed and low physical activity) [24]. The other widely-used approach conceptualizes frailty as the result of multiple interacting factors, to create an index as a proportion of deficits [25], [26]. Deficit accumulation or frailty indices can be constructed from different numbers and types of variables, allowing comparisons between datasets [27]. For example, analysis of data for 36, 424 older people in four developed countries found frailty index values to be closely comparable across countries, increasing with age at approximately 3% per year in community-dwellers and correlating highly with mortality [28]. The Frailty Index approach has also recently been adopted by developing countries, exploring the affect of health status on type of death [29]. Further studies confirm that the risk of adverse outcomes is defined more precisely by deficit indices than by phenotypic definitions of frailty [30].

As in earlier reports, [31], [32] this frailty index was determined from 40 variables, selected as representing a range of health conditions and disabilities. No variable had more than 5% missing values; where missing values existed, they were inputed using the relevant mean [33]. Each variable represents a potential health deficit (e.g. symptoms, signs, functional impairments, co-morbidities, poor health attitudes). For any individual, the Frailty Index is the number of deficits present, divided by the number of deficits counted, here 40: hence someone with 6 deficits would have a Frailty Index of 0.15.

With respect to clinical translation, the Frailty Index can capture gradations in health status and the risk of adverse outcomes. It has been contextualised against a Clinical Frailty Scale in 2305 participants of the Canadian Study of Health and Ageing; this describes the functional and clinical characteristics related to different Frailty Index scores [34]. For example, those who are well with treated co-morbid disease have a mean of 6.4 deficits out of 40 (FI 0.16) whereas an FI of 0.36 (14 deficits) tends to describe those who need help with both instrumental and non-instrumental activities of daily living [31]. Note too that most people with more than 9 deficits out of 40 (Frailty Index score >0.22) are frail by any definition [34], [35].

### Analysis

To distinguish the impact of exercise in relation to graded exposures (i.e. the different levels of health graded in the frailty index) with four different outcomes (improved health status, same status, worse status or death) we employed a multi-state model [36], [37], [38] (Appendix S1). The model allows all possible outcomes at all relevant health states to be summarized with just four parameters, and for the influence of co-variates (age, sex) on these parameters to be estimated. To minimize the inaccuracies of predicting outcomes for very small numbers of participants, 23 people with 18 or more deficits (i.e. Frailty Index scores >0.45) were combined in a single group.

## Results

We have complete data, including data for frailty status and exercise participation at baseline as well as frailty status or death at 5 years, on 5555 participants (Figure 1). Compared to the low/no exercisers, the 2708 participating in regular exercise tended to be younger and comprised a higher proportion of men. The high exercise group was significantly fitter than low/no exercisers, with a mean Frailty Index (FI) values of 0.08 (SD 0.06) compared to 0.11 (0.09) (Table 1).

**Table 1:** Demographics, mean frailty index and mean 3MS cognitive scores.

	High Exercise n = 2708	Low/No Exercise n = 2847	Non Respondents at CSHA-2 n = 742
Age, mean (SD)	73.5 (6.2)	75.3 (6.8)	75.4 (6.8)
Exercise (%)	100	0	45.9
Sex (% female)	54.2	63.0	63.8
3MS total score, mean (SD)	91.1 (5.9)	89.7 (6.0)	88.0 (6.0)
Frailty Index at CSHA-1, mean (SD)	0.08(0.06)	0.11(0.09)	0.10 (0.08)

As one might expect, baseline frailty and participation in little or no exercise were each associated with an increased risk of death. Using logistic regression techniques, the risk ratio for frailty was 1.21 (95% CI 1.19 to 1.24) and for low exercise 1.95 (1.73 to 2.28).

**Figure 2:**
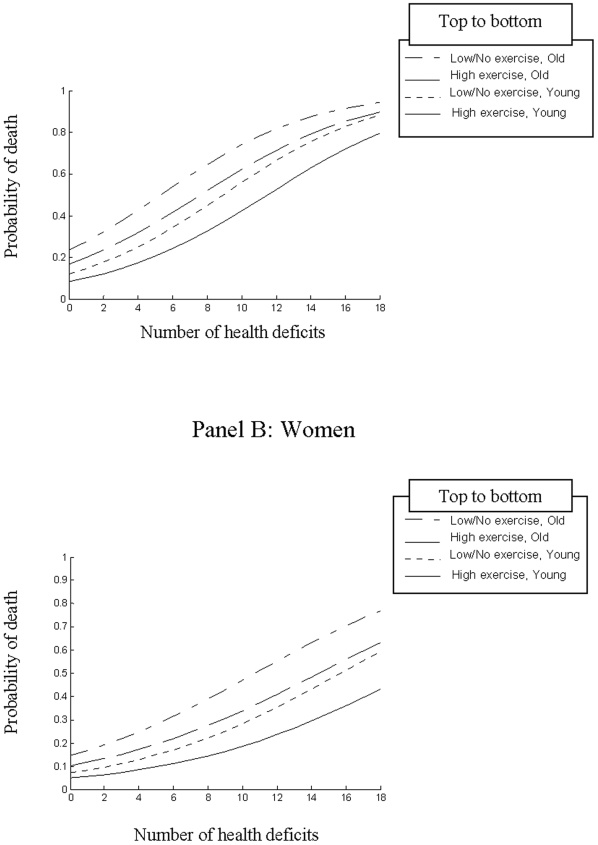
Probability of death within 5 years by number of deficits at baseline with participants grouped by age (<75 years, ≥75 years) and exercise status (high exercise: three or more times per week, at least as intense as walking or low/no exercise: all other exercisers and non exercisers). Panel A: Men, Panel B: Women.

Exercise had an impact on both mortality and on health status that was highly fit with a Markov model (Appendix S1). For both men and women, whether older or younger, mortality increased as number of health deficits at baseline increased (Figure 2). The effect of exercise was to attenuate the impact of age on mortality i.e. for both men and women; those aged ≥75 years who exercised had a similar probability of death to those aged <75 who did no exercise. While exercise reduced the risk of death in all participants, it conferred its greatest mortality benefit in those with lower baseline frailty. For example, using unadjusted data the relative risk of death for low exercisers was 2.39 (95% CI 1.18–4.81) for the fittest older people (with 0 deficits at baseline) compared to a relative risk of 2.11 (0.92–4.77) for older people who would be considered frail (those with 9 deficits at baseline, a Frailty Index of 0.225).

**Figure 3:**
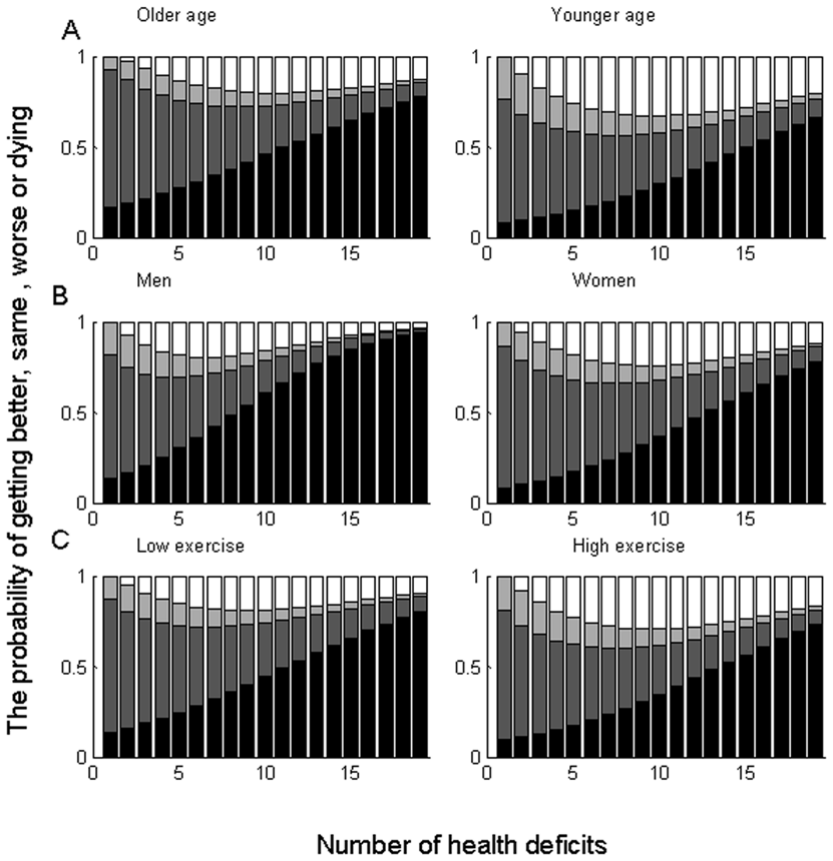
The probability of getting better (beige), remaining the same (tan), getting worse (dark brown) and mortality (black) as a function of number of health deficits at baseline. Panel A: <75 years vs. ≥75 years Panel B: men vs. women Panel C: low/no exercise vs. high exercise.

With respect to changes in health status, and noting that people with 0 deficits at baseline have no opportunity to improve, there was no reduction of benefit across the frailty states studied. Rather, all those who exercised had a greater chance of improving their health status, which was enhanced as baseline frailty worsened (Figure 3). For example, improvement or stability was observed in 34% of high exercisers versus 26% of low/no exercisers for those with 2 deficits (FI 0.05) compared with 40% of high exercisers versus 22% of low/no exercisers for those with 9 deficits at baseline (FI 0.225).

## Discussion

In this secondary analysis of the Canadian Study of Health and Aging, we evaluated the impact of exercise on health status and near term (up to five year) survival of older people. We found that people who participated in high levels of physical activity had a lower risk of death than those who did little or no exercise. Death rates for both men and women aged over 75 who exercised were similar to their peers aged 65 to 75 who did not exercise. By mapping the transitions in numbers of health deficits in relation to exercise and adjusting for age and sex, we found that people who exercised had a greater chance of improving their health status than those who did not exercise. Interestingly, the absolute benefits in health status were greatest for those with the highest number of health deficits, *i.e*. the most frail.

Our data must be interpreted with caution. First, the measurement of frailty is an area of ongoing debate and the definition of frailty used here is not the only one available [22]. Phenotypic definitions of frailty tend to dichotomise or trichotomise participants (e.g. “frail”, “pre-frail” or “non frail” [24]) and may exclude many of the “frailest” participants who are unable to complete performance based tests [39]. Here we were interested in quantifying risks of death and in capturing subtle changes in health status across the whole health continuum. The use of the deficit count therefore seems most appropriate for this study. Second, the follow up period was only 5 years and the effects of exercise on health transitions needs to be examined for longer follow up periods. Note too that we used self reported data. Self-report of physical activity has limitations and correlation with objective assessment is variable [40]. On the other hand, more objective physical performance measures tend to under-estimate the impact of poor function, as they commonly exclude people with the worst performance [41] and by using broad groups of activity levels, we minimize the impact of self-reporting bias. Finally, although the CSHA is a large, representative sample, 11.8% of eligible participants were lost to follow up. Since these non respondents did not appear to be systematically different to those remaining in the study at CSHA-2, this is unlikely to have materially affected our conclusions.

These results cannot be extrapolated to those in residential or nursing homes or to those with significant cognitive impairment. In this study, we investigated only community-dwelling older people who were able to complete a self-administered questionnaire. CSHA participants unable to do so were older (mean age 78.5 y [SD 7.4]) and more likely to be cognitively impaired (mean 3MS score 75.2 [15.1]). However, in longitudinal cohort studies low exercise is a risk factor for dementia [42] and analysis of this CSHA cohort showed exercise to be strongly associated with improving cognition [38]. In a recent randomized controlled trial of adults with subjective memory impairment, a 6-month program of physical activity provided a modest improvement in cognition over an 18-month follow-up period [43] and cognitively impaired older adults who participate in exercise rehabilitation programs have similar strength and endurance training outcomes as age and gender matched cognitively intact older participants [44]. There is therefore reason to be optimistic that the benefits of exercise do extend to those with cognitive impairment at baseline.

Our results suggest that older people benefit from exercise with lower mortality rates and increased likelihood of improvement in health status. These benefits extend to those with higher numbers of health deficits at baseline. Our study provides some evidence to relieve the concern that health prevention in older people extends longevity by prolonging time in impaired states. Exercise conferred its greatest benefits to improvements in health status to those who were more frail. At a population level, age and frailty should be reasons to promote rather than to limit physical activity.

## Supporting Information

Appendix S1 Technical Appendix for the statistical reviewer
(0.04 MB DOC)
