# Supplementary material for: Impact of Exercise in Community-Dwelling Older Adults
Source: PLoS One. 2009 Jul 8;4(7):e6174. doi: 10.1371/journal.pone.0006174 (PMC2702830; doi:10.1371/journal.pone.0006174)
Supplement: Appendix S1 — Technical Appendix for the statistical reviewer (0.04 MB DOC) [file pone.0006174.s001.doc]

APPENDIX

**The modified Poisson model**

We used the following stochastic model to describe changes in individual health status. Given any individual’s initial frailty state as ‘n’, let $P_{nk}$ be the probability that this individual will have frailty state ‘k’ at the time of the next assessment, and let $P_{nd}$ be the probability of dying before the next assessment. When the number of states is large, (~>10) the transition probabilities between the different numbers of states can be approximated by a modified Poisson distribution. Here we use a Poisson distribution to represent the transition probabilities when the number of states, N, is finite:

$P_{nk}=\frac{\rho_{n}^{k}}{k!}e^{-\rho_{n}}(1-P_{nd})$ (A1)

The term 1-P_nd_ is the probability of survival between two assessments. In other words, for each n, the transition probabilities satisfy a Poisson distribution in which the parameter $\rho_{n}$ depends on the current state n as follows:

*ρ_n_=α_1_+β_1_n* (A2)

The interpretation of the parameters α_1_ and β_1_ is as follows: *α*_1_ is *ρ*_0_ (it is the mean state number k given the zero state at baseline, i.e. n=0). The *β*_1_ is the state increment when

n > 0.

The Poisson parameter can also depend on age, sex, education, or other conditions. Considering a set of m binary covariates z_j_  we use the following representation of the Poisson parameter *ρ* :

$\rho(n,z_{1},z_{2},...)=\alpha_{1}+\beta_{1}n+\sum_{j=1}^{m} \gamma_{1j}z_{j}+\sum_{j=1}^{m} \delta_{1j}z_{j}n$ (A3)

The probability of death can also be parameterized as following:

*logit*(*P*_nd_)$=\alpha_{2}+\beta_{2}n+\sum_{j=1}^{m} \gamma_{2j}z_{j}+\sum_{j=1}^{m} \delta_{2j}z_{j}n$ (A4)

Based on equations (A1 –A4) the likelihood function can be written:

$L=\prod_{i=1}^{M_{a}} (\alpha_{1}+\beta_{1}n+\sum_{j=1}^{m} \gamma_{1j}z_{j}+\sum_{j=1}^{m} \delta_{1j}z_{j}n)^{k_{i}} e^{-(\alpha_{1}+\beta_{1}n+\sum_{j=1}^{m} \gamma_{1j}z_{j}+\sum_{j=1}^{m} \delta_{1j}z_{j}n)}1/[1+e^{(\alpha_{2}+\beta_{2}n+\sum_{j=1}^{m} \gamma_{2j}z_{j}+\sum_{j=1}^{m} \delta_{2j}z_{j}n)}]\times$ $\prod_{i=1}^{M_{d}} e^{(\alpha_{2}+\beta_{2}n+\sum_{j=1}^{m} \gamma_{2j}z_{j}+\sum_{j=1}^{m} \delta_{2j}z_{j}n)}/[1+e^{(\alpha_{2}+\beta_{2}n+\sum_{j=1}^{m} \gamma_{2j}z_{j}+\sum_{j=1}^{m} \delta_{2j}z_{j}n)}]$ (A5)

where index i-refers to the i-th person, M_a_ is the number of survivors and M_d_ is the number of people who died within the period of assessment.

**Estimation of the parameters**

The parameters of the model (***α****,****β****,****γ****,****δ***) are estimated by maximizing the likelihood function (A5) using procedures “fminsearch” and “fminunc” from optimization toolbox in Matlab (Matsft Inc.) The function fminsearch is an implementation of Nelder-Mead simplex method. The function fminunc allows different algorithms to be toggled, including Newton’s method (if gradient is provided) and BFGS otherwise. The procedure also calculates the Hessian matrix used for the computation of the standard errors of the parameters. The codes are available for academic user upon request.
